# Establishment of health related physical fitness evaluation system for school adolescents aged 12–16 in Pakistan: a cross-sectional study

**DOI:** 10.3389/fpubh.2023.1212396

**Published:** 2023-09-27

**Authors:** Syed Muhammad Zeeshan Haider Hamdani, Jie Zhuang, Syed Ghufran Hadier, Haris Khurram, Syed Danish Haider Hamdani, Shaista Shireen Danish, Syeda Urooj Fatima, Wang Tian

**Affiliations:** ^1^Faculty of Sport Science, School of Kinesiology, Shanghai University of Sport, Shanghai, China; ^2^Faculty of Arts and Social Sciences Department of Sports Sciences, Bahauddin Zakariya University, Multan, Punjab, Pakistan; ^3^School of Physical Education, Shanxi University, Taiyuan, Shanxi Province, China; ^4^Department of Sciences and Humanities, National University of Computer and Emerging Sciences, Islamabad, Pakistan; ^5^School Education Department, Government of Punjab, Lahore, Pakistan; ^6^Department of Physical Education, Government College University Faisalabad, Faisalabad, Pakistan

**Keywords:** physiological testing, cardiorespiratory fitness, muscle strength, public health, evaluation system, protocol and measurement method

## Abstract

**Background:**

The decline in adolescent physical fitness is a significant global public health concern, and Pakistan is no exception. The country’s absence of a health-related physical fitness (HRPF) evaluation system has compounded this issue. To bridge this gap, this study aims to develop a scientifically-based HRPF evaluation system for the adolescent population that meets international standards. The evaluation system identifies at-risk children and improves adolescent health outcomes, including obesity, cardiovascular and musculoskeletal disorders, chronic diseases, and psychological illnesses, through crucial physical fitness evaluation. This study specifically aims to establish an HRPF evaluation system for school adolescents aged 12–16 in Pakistan.

**Methods:**

A cross-sectional study was conducted among 2,970 school adolescents aged 12–16 years in the South Punjab, Pakistan. The study used a stratified sampling technique to select participants. The HRPF evaluation system included four components: cardiorespiratory endurance, core muscular endurance, muscular strength, and body composition. Data were collected through standardized tests and anthropometric measurements.

**Results:**

The study’s results indicated that the HRPF evaluation scoring system was feasible and valid for evaluating the HRPF of school adolescents in the South Punjab region of Pakistan. The results of the evaluation system categorized participants into five groups based on their performance: excellent (6.2%), good (24.9%), medium (50.7%), poor (17%), and very poor (1.2%).

**Conclusion:**

The study establishes an HRPF evaluation system for Pakistani school adolescents. This system lays the foundation for implementing effective strategies to improve their physical health. The findings offer valuable insights to policymakers, health professionals, and educators, enabling them to promote fitness and devise impactful interventions for enhancing HRPF in this population.

## 1. Introduction

The rapid development of the economy, urbanization, and changes in modern life have contributed to the emergence of various diseases (1). One such disease is obesity, which is increasingly prevalent among adolescents worldwide (2). Additionally, global variations in human growth at different ages are essential to identifying the factors that contribute to this trend, including genetics, diet, physical activity (PA) levels, physical fitness (PF), and socioeconomic variables (3). The decline of adolescent PF has emerged as another global concern threatening human health (4). PF is a crucial indicator of an individual’s overall health. It is associated with a reduced risk of chronic diseases, such as cardiovascular disease, type 2 diabetes, and certain types of cancer (5). In the late 19th century, many nations began recognizing health-related physical fitness (HRPF) research as a critical indicator of an individual’s overall health and well-being (6. p4). Consequently, assessing PF and its relationship to health became a top priority for promoting the health of children and adolescents (7). As a result of the pressing health issues faced by adolescent populations, many countries placed great importance on HRPF research as a means of solving these problems (8).

Health-related physical fitness reference standards provide a way to measure and track changes in fitness levels over time and identify individuals at risk of health problems related to low PF (9). HRPF reference standards are essential for identifying and managing the impact of health problems related to physical inactivity and sedentary behavior (SB) among adolescents (10). Growth and PF reference standards identify at-risk individuals and can also be used to monitor the effectiveness of interventions to address these children and adolescents’ health issues, such as obesity (11, 12). Furthermore, reference standards of different anthropometric measures provide a benchmark for measuring changes in height, weight, and body mass index (BMI) over time, allowing for the identification of individuals at risk of health problems related to under or over-nutrition (13). HRPF reference standards are vital for research and clinical practice as they provide a standardized basis for diagnosing and managing growth disorders, such as short stature or delayed puberty. The reference standards also allow researchers to compare PF levels across different populations and identify existing disparities over time (14).

Researchers and policymakers are working to guide school-aged adolescents toward engaging in fitness activities based on scientifically-informed practices to promote overall health. This information can help in the development of effective health promotion and disease prevention programs, target high-risk populations, and monitor changes in health status over time (15). The measurement and evaluation of PF in adolescents have become a significant area of focus in sports science research as they provide valuable information about the current state of PF and health among adolescent populations (16). Adolescents are a critical population to monitor regarding PF and health, as this is a time of rapid physical and emotional development that can have a lasting impact on overall health and well-being (17, 18). PF evaluation systems for adolescents typically measure several vital components of physical fitness, including cardiovascular endurance, muscular strength and endurance, and body composition (19).

Over the years, numerous countries have conducted extensive theoretical and experimental research on HRPF among adolescents; these researchers have identified specific characteristics and trends in the field, including unique aspects of HRPF in adolescents that must be considered when developing appropriate measurement and evaluation tools (20). Despite these differences, there is a shared trend toward increasing scientific rigor and precision in the measure of HRPF in order to better guide and promote health among school-aged children. This trend underscores the importance of adopting a standardized and evidence-based approach to assessing HRPF, which can lead to more effective interventions and better health outcomes.

The need for HRPF evaluation systems is contingent upon various factors across different regions of the world, including regional variations in disease prevalence, cultural and socioeconomic influences, and access to healthcare (21). PF evaluation systems for adolescents serve various essential public health purposes in different regions. One important use of these systems is to monitor and identify disparities in health by comparing PF levels across other regions and demographic groups around the globe. By detecting disparities in health, PF evaluation systems can provide a basis for developing targeted public health interventions (22).

Moreover, PF evaluation systems are essential for developing health promotion programs; the information obtained from these systems can be used to design and evaluate programs to improve PF and reduce the risk of chronic diseases (23). The global burden of chronic diseases, including heart disease, diabetes, and obesity, is increasing, making it necessary to use PF evaluation systems to address this trend (24). These systems can play a vital role in assessing PF to reduce the global burden of chronic diseases by providing insight into risk factors and guiding the development of effective public health interventions.

The global prevalence of obesity, SB, physical inactivity, and low level of HRPF among adolescents, particularly in developing countries like Pakistan, is a complex health problem (25, 26). However, limited previous research has been found to focus on these health issues, with few studies examining PF, PA, and SB among adolescents in Pakistan (25, 27, 28). Furthermore, previous studies were limited by factors such as sample size, age differences, socioeconomic disparities, and other potential confounding variables that may affect the results (28, 29). Therefore, it is urgent to strengthen the research on the evaluation and measurement of the health-related physical fitness of adolescents in Pakistan.

Thus, the objective of this study is to establish a health-related physical fitness evaluation system of key health-related physical fitness indicators, including hand-grip strength (HG), modified pull-up (MPU), plank endurance (PE), and 20-m shuttle run (VO2max), among school adolescents aged 12–16 in the South Punjab region of Pakistan. This evaluation system will employ globally recognized assessment indices for anthropometric characteristics, obesity, and internal physiological mechanisms such as muscular strength, cardiovascular fitness, endurance, and cardiorespiratory fitness. By doing so, this study aims to address the gaps in the literature and provide a scientific and logical based comprehensive evaluation of the HRPF of adolescents in the South Punjab region.

## 2. Materials and methods

The present cross-sectional study aimed to establish an evaluation system of health-related physical fitness indicators among adolescents in South Punjab, Pakistan. This study was conducted as part of the Young teen’s Assessment Active Lifestyle Involvement—PAKistan Study (YAALI-Pak) (28), which seeks to understand the lifestyle habits and associated health outcomes of Pakistani adolescents.

A panel of nine experts in sports science and physical education in Pakistan was invited to provide an opinion on the development process of the adolescent HRPF evaluation system for the South Punjab region. The panel offered consensus-based opinions on the inclusion of indicators and weightage for each measure, tailoring the evaluation system to the unique characteristics and needs of the region. The experts expressed satisfaction with the adherence to the protocols established by the WHO and CDC for conducting measurement methods and physiological tests (6, 14, 30). The HRPF evaluation system meets the highest standards for measuring and monitoring adolescent physical fitness in the region.

### 2.1. Sampling and sample size

The researchers employed a stratified random sampling method to obtain a representative sample. The South Punjab region was used as the stratum and further divided into three sub-strata: Multan, Bahawalpur, and D.G. Khan. The majority of the populations of these stratums are a good representation of the Southern Punjab population. So, these areas/regions contain the representative population of South Punjab. From 360 higher schools in the region, 20 higher schools were randomly selected from each stratum using an equal allocation method, making up 16.67% (60 higher schools) of the total schools in the region. A general sample of 50 students from each school was taken/selected. The total sample size was 3,000, as shown in Table 1. Finally, 2,970 respondents were included in the study after removing non-responsive or incomplete respondents, which was about 1%.

**Table 1:** List of indicators included in the health-related physical fitness evaluation system.

Test category	Indicators	Name
Body composition	Body figure	BMI (kg/m^2^)
Health-related physical fitness	Upper body muscular strength	Hand grip (kg)
	Muscular strength	Modified pull-up (*n*)
	Core muscular strength and endurance	Plank exercise (s)
	Aerobic capacity	20 m shuttle run test (laps)

Moreover, the sample size power of the test was greater than 80%, and sample size calculators and formulas provide sample size at a 5% level of significance with a 95% precision level lower than this sample size (28, 31). The current study was conducted in south Punjab, including three divisions: Multan, Bahawalpur, and D. G. Khan. A total of 60 schools, with 20 schools from each division, participated in the study. The sample size included 3,000 participants aged between 12 and 16 years, with 50 students randomly selected from each school. The findings of this study can be generalized to the population of school-going adolescents aged 12–16 years in the South Punjab region. After removing 30 non-responsive participants, a total of 2,970 school adolescents (49.73% boys and 50.26% girls) were included in the final analysis. The majority of the populations of these stratums are a good representation of the Southern Punjab population. Only physically and mentally healthy adolescents with no prior injury record or intention to receive monetary compensation were included in the study. Workshops were conducted to inform relevant parties, including the school management, parents, test and measurement staff, and students, about the study project and testing procedures.

### 2.2. Ethical approval

The Shanghai University of Sport’s School of Exercise and Health, along with its Ethics Advisory Committee, approved the research design and methodology in September 2018 the study approval number (1716516032). To uphold ethical standards, the study obtained written and verbal consent from relevant authorities, including educational authorities, school management, and parents, prior to conducting sampling and testing procedures. Data collection took place in 2019 after receiving approval from the education authorities. These precautions ensured the protection of participants and adherence to institutional regulations. The study adheres to all ethical standards and complies with the Declaration of Helsinki.

### 2.3. Variable and description

#### 2.3.1. Determination of indicators for health-related physical fitness evaluation system

From the perspective of the development of the health-related physical fitness evaluation system, all national and international groups working on developing or implementing their evaluation system strive for consensus on how to interpret the concept of building and which evaluation indicators should be used for field-based tests. This study chose HRPF indicators like muscular strength, muscular endurance, and cardiorespiratory fitness among adolescents in Pakistan based on consultation with subject-matter experts and a thorough review of the aforementioned global trends in health-related physical fitness research. In order to create a scientific and systematized system for detecting HRPF, it is necessary to choose a specific number of indicators that can more truly reflect the fitness development characteristics of adolescents. This will allow each indicator to reflect a particular physical characteristic of a given age accurately. The following indicators were used in the current study to achieve the objectives mentioned above: body composition (BMI), muscle strength (Hand-grip/Modified pull-up), cardiopulmonary fitness (20-m shuttle run), and muscle endurance (Plank; Table 1).

#### 2.3.2. Test methods, procedures

The study followed a rigorous protocol for HRPF indicators, modeled according to the National Adolescents Fitness Survey (NYFS-2012) by the Centers for Disease Control and Prevention in the United States (32). To ensure the accuracy and consistency of the results, the subjects were given ample time to warm up before the physical fitness tests were conducted. Additionally, the students were advised to perform cool-down exercises after the tests to minimize the risk of any potential injuries. Detailed measurement procedures can be found in previous studies (28, 32).

#### 2.3.3. Data collection and schedule

Data were collected during the 2019 academic year across schools in the South Punjab Region, Pakistan. School visit schedules were formulated around school timetables. Two days prior to each visit, the test team communicated with school personnel to communicate test requirements (33). On the day of the visit, the researcher randomly chose students for data recording. Only these selected students constituted the study sample. The class teacher assured their presence for subsequent visits (34).

##### 2.3.3.1. Testing protocol

The testing procedure spanned 3 consecutive days at each school during regular hours:

###### 2.3.3.1.1. First day

Anthropometric data were gathered, including height, weight, and specific body measurements (32). A plank exercise was also conducted for core muscular endurance (33). Later, participants filled out the International Physical Activity Questionnaire (short form).

###### 2.3.3.1.2. Second day

Physical fitness tests measuring strength, like handgrip strength and modified pull-ups were conducted (34).

###### 2.3.3.1.3. Third day

Tests assessing aerobic capacity, such as the Shuttle Run Test (20 m), performed (28).

To ensure engaged participation, the research team interspersed fun activities amidst the tests (34).

###### 2.3.3.1.4. Warm-up and cool-down

Before each physical test, participants underwent a 10-min standard warm-up consisting of light aerobic exercises and dynamic stretches to prepare their bodies. Post-test, a 5-min cool-down of slow walking and static stretches was advised to minimize injury risks (32).

#### 2.3.4. Selected anthropometric parameters and body mass index

The individuals’ anthropometric characteristics were measured per the procedures outlined in the literature. The measurements were taken while the individuals were barefoot and wearing lightweight clothing to prevent inaccurate readings. Height was measured using a height-measuring scale, and weight was measured using a portable digital weight machine. Body mass index (BMI) was then calculated using height and weight values (32).

#### 2.3.5. Health-related physical fitness measures

Health-related physical fitness indicators are important because they provide a measurement of an individual’s physical health and ability to perform physical tasks. To assess these indicators (cardiovascular endurance, cardiorespiratory fitness, muscular strength, and endurance), one should choose a specific test for each consistent and effective indicator in a particular research environment. As part of the YAALI-PAK study, detailed information regarding measurement tools and test procedures has already been published (28).

#### 2.3.6. Core muscular endurance plank exercise

The Plank exercise is an isometric test to evaluate the muscles’ endurance in the body’s core region (33). Test Method: To perform the Plank test, the participant assumes a prone position with their elbows bent, shoulders and elbows perpendicular to the ground, and feet on the ground. They lift their body off the ground, keeping their trunk straight and tightening their abdominal and pelvic floor muscles. The tester times the duration of the hold with a stopwatch, and the test ends if the participant deviates from the standard position. The recorded time in seconds is then reported to the recorder.

#### 2.3.7. Upper body muscular strength hand grip

Grip strength is a commonly used measure of upper limb muscle strength and reflects an adolescent’s level of upper limb development (34). The GRIPX Digital Hand Dynamometer Grip Strength Measurement is a standard device used to measure hand-grip strength in this study. Before testing, the handle distance is adjusted to the palm size using the handle adjustment knob. During testing, the subject stands with feet naturally separated, shoulders adducted, and arms neutrally rotated, with arms naturally opened 30 degrees’ parallel to the body interface but not touching it. The subject is instructed to squeeze the handle for 3–5 s, and the numeric result is displayed on the device’s LCD screen and recorded by the recorder.

#### 2.3.8. Muscular strength modified pull-up

The Modified Pull-Up (MPU) test is a hanging strength assessment used to measure muscular strength in the body, particularly in the back muscles. The test involves using a modified horizontal bar and a gymnastics mat. The bar is adjusted to an appropriate height and positioned on a flat surface, and a nylon strap hangs 8 in down from the center of the bar. During the test, the participant hooks the bar with an overhand grip of both hands, palms facing away from the body, and keeps their body straight with arms at a 90-degree angle to the trunk. The participant then bends their arms to make their chin touch or exceed the crossbar and straightens their arms to recover. The number of correctly completed pull-ups is recorded, and the test ends when the participant pauses for two or more seconds, cannot maintain the correct position, or requests to stop. Scores are based on the number of correctly completed pull-ups.

#### 2.3.9. Cardiorespiratory fitness /aerobic capacity 20 m shuttle Run test

The Shuttle Run Test 20 m assessed the participants’ aerobic capacity by measuring the time to run back and forth across a marked 20 m track while keeping up with the beeps at increasing speeds. The test began at a speed of 8.0 km/h and increased by 0.5 km/h each minute. Participants were required to cross the finish line before the beep sounded, and failure to do so twice resulted in disqualification. The recorder recorded the completed laps as the final result.

The chosen methods and tests ensure a holistic assessment of adolescent health and fitness (28). They were selected for their precision, reliability, and alignment with established standards in adolescent health research (32).

### 2.4. Statistical analysis and evaluation system

The present study comprehensively analyzed the adolescent population’s anthropometric and health-related physical fitness indicators. Descriptive analysis and inferential statistics were calculated, such as percentages, frequencies, mean, and standard deviation. To obtain normative reference values and LMS curves using the Lambda (λ), Mu (μ), and Sigma (σ) methods (35). The LMS method was applied using R statistical software version 3.0.2 to generate LMS curves for age-specific and gender-specific indicators. The normative reference values obtained through the LMS method were used to draw indexes for individual health-related physical fitness indicators. The back substitution test approach was applied to ensure the standard’s cross-validation and applicability. The study used a consensus-based approach to establish indicators and their weightage for a comprehensive evaluation system of health-related physical fitness in adolescents aged 12–16. The experts’ input led to criteria for both genders, resulting in practical and relevant evaluation. Incorporating scientific principles and methods in this study enhances its credibility and applicability.

## 3. Results

Table 2 shows the population’s age and gender-specific demographic descriptive analysis. The current study testified a total of 2,970 participants aged 12–16 years’ school adolescents. Boys and girls were distributed equally as 1,477 (49.7%) and 1,493 (50.3%). Further, around 20% of the population was distributed from each age group with each gender.

**Table 2:** Age and gender-specific frequency analysis *N* (%).

Age (years)	Boys	Girls	Total
12	291 (19.7%)	299 (20.24%)	590 (19.9%)
13	295 (19.97%)	298 (20.17%)	593 (20.0%)
14	298 (20.17%)	296 (20.04%)	594 (20.0%)
15	298 (20.17%)	300 (20.31%)	598 (20.1%)
16	295 (19.7%)	300 (20.31%)	595 (20.0%)
Total	1,477 (100%)	1,493 (100%)	2,970 (100%)

**Figure 1:**
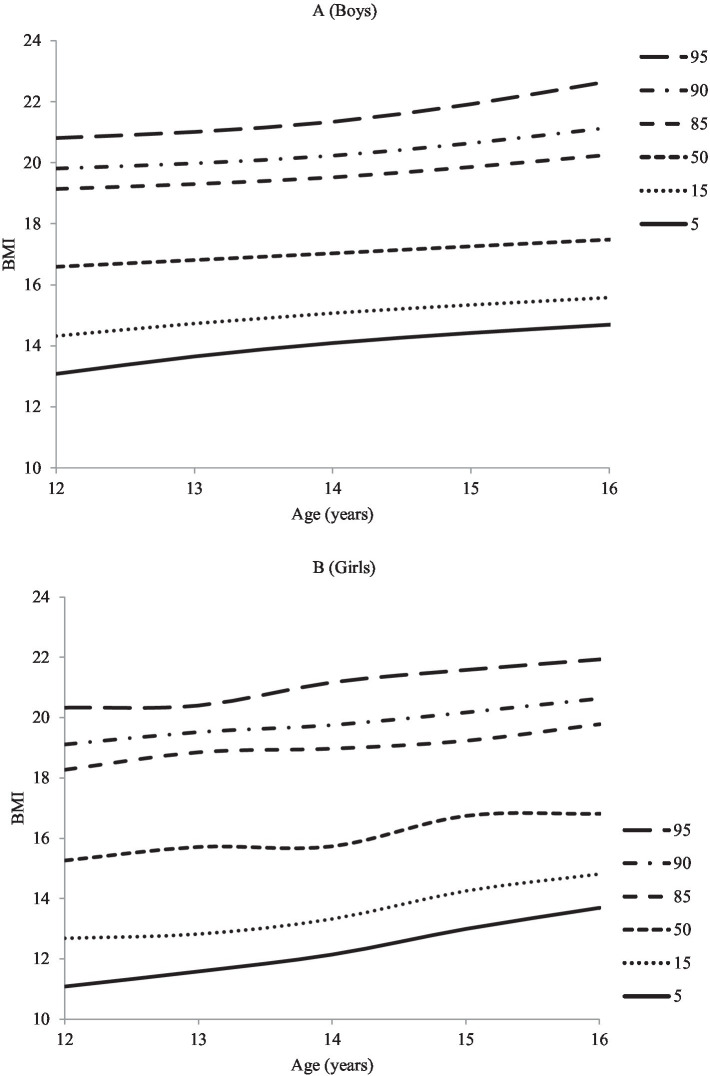
BMI (kg/m^2^) smoothed percentile curves for South Punjab **(A)** Boys and **(B)** Girls.

After numerous adjustments and verifications, the current study used the LMS method to develop reference values and percentile curves for adolescents of different ages. The obtained reference values and percentile curves for both boys and girls are presented in Table 3 and Figure 1. The median (P50th) centiles for body mass index (BMI) in boys and girls aged 12–16 years ranged from 16.59 to 17.48 and 15.71 to 16.81, respectively. The median (P50th) BMI for boys increased by 0.89, while for girls, it increased by 1.1. After analyzing the trend and variation of BMI in boys and girls showed a negligible change over the course of 1 year (approximately). Overall, male participants had higher BMI percentile values compared to female participants.

**Table 3:** Age and gender-specific BMI percentile generated by the LMS method.

Age	L	M	S	5	15	50	85	90	95
Boys									
12	0.203	16.59	0.141	13.08	14.32	16.59	19.14	19.81	20.81
13	−0.318	16.81	0.131	13.65	14.73	16.81	19.30	19.98	21.01
14	−0.838	17.03	0.125	14.09	15.07	17.03	19.52	20.23	21.34
15	−1.358	17.26	0.124	14.42	15.34	17.26	19.86	20.64	21.92
16	−1.879	17.48	0.125	14.69	15.58	17.48	20.26	21.15	22.67
Girls									
12	0.283	15.26	0.202	11.08	12.68	15.26	18.27	19.11	20.33
13	−0.173	15.71	0.172	11.58	12.82	15.71	18.85	19.52	20.40
14	−0.495	15.73	0.168	12.14	13.32	15.73	18.97	19.75	21.17
15	−0.234	16.74	0.159	12.99	14.25	16.74	19.23	20.17	21.58
16	0.382	16.81	0.120	13.69	14.81	16.81	19.78	20.63	21.93

L (γ-lambda), M (μ-mu), and S (δ-sigma) algorithm.

**Figure 2:**
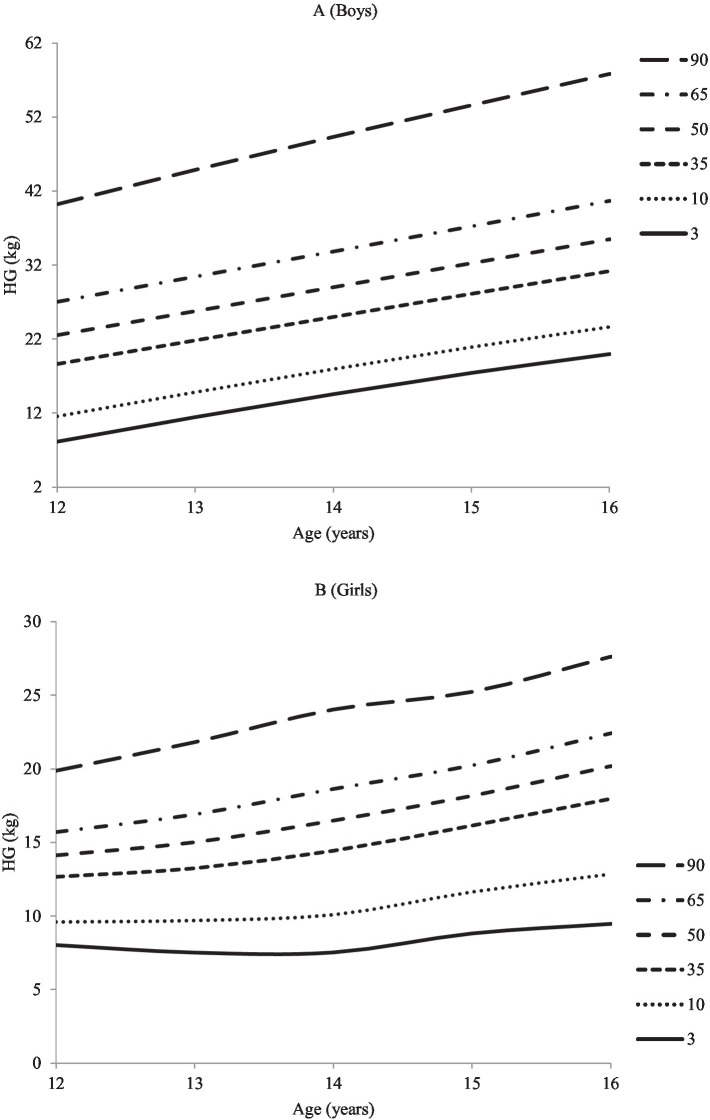
Hand grip strength (kg) percentile curve for the adolescents **(A)** Boys and **(B)** Girls.

Additionally, the study employed the LMS method to establish reference values and percentile curves for hand-grip strength in adolescents of both genders. After several adjustments and verifications, the study obtained the hand-grip strength values for boys and girls of different ages, presented in Table 4 and Figure 2. The Smoothed LMS curves (for the 3rd, 10th, 35th, 50th, 65th, and 90th centiles) for the hand-grip strength of boys and girls are also shown in Figure 2, while the corresponding numerical data are provided in Table 4. The results indicate that boys have better hand-grip strength test scores than girls across all age groups, as evidenced by the centiles. The 50th centile value for hand-grip strength for boys and girls ranges from 22.53 to 35.47 and 13.12 to 20.18 kg, respectively.

**Table 4:** Hand grip indicators men and women P 50th measured value.

Percentile	L	M	S	3	10	35	50	65	90
Boys									
12	0.227	22.53	0.483	8.14	11.56	18.63	22.53	27.03	40.21
13	−0.004	25.77	0.432	11.45	14.82	21.82	25.77	30.43	44.86
14	−0.198	29.00	0.393	14.55	17.94	24.98	29.00	33.82	49.31
15	−0.339	32.24	0.364	17.42	20.90	28.11	32.24	37.22	53.58
16	−0.413	35.47	0.345	19.96	23.63	31.17	35.47	40.66	57.81
Girls									
12	0.268	14.12	0.279	8.02	9.59	12.66	14.12	15.70	19.87
13	0.442	15.01	0.317	7.51	9.69	13.24	15.01	16.90	21.81
14	0.615	16.48	0.331	7.52	10.08	14.43	16.48	18.63	24.03
15	0.789	18.16	0.293	8.81	11.63	16.14	18.16	20.23	25.23
16	0.963	20.18	0.286	9.46	12.84	17.96	20.18	22.41	27.62

L (γ-lambda), M (μ-mu), and S (δ-sigma) algorithm.

Additionally, an average annual increase of approximately 3–5 kg was observed in boys and girls. The study also found that muscle strength increases with age in both boys and girls, with a marked increase between the ages of 14 and 15. Overall, the results presented in Table 4 indicate that boys have a higher hand-grip strength percentile than girls.

The back substitution test is a crucial step in evaluating the cross-validation of an evaluation system. This study involves randomly extracting a small data set from a larger one to assess the consistency between the fitted and actual values. In our study, we used the LMS method to establish reference norms for HRPF indicators and identify the absolute mean error rate by comparing the p50 values of the large (actual value) and small (fitted values). The back substitution process assessed the degree of coincidence between data sets generated using a random sampling method to test the cross-validation of the standard. By conducting the back substitution test, we ensured that a lower error rate indicated higher accuracy for our evaluation system. In this study, we employed back substitution and evaluated one index from the body shape and health-related physical fitness indicator. This back-substitution approach ensured the standard was based on valid scientific principles, increasing its credibility and applicability.

Table 5 presents an example of the back substitution test used to determine body composition indicators. This process involves adopting the 50th percentile of the BMI fitted value and comparing it to the actual value in the data. The results show that the absolute difference between the actual and fitted values of each age group is generally within the allowable mean absolute error rate of the measurement. This finding indicates that the actual and fitted values of absolute mean error is within an acceptable range across the 12 to the 16-year-old age group for both genders.

**Table 5:** Back substitution test of BMI, Hand grip age, and gender-specific comparison of P 50th measured value with the fitted value (kg).

Age	Male			Female		
	Actual value	Fitted value	MAPE	Actual value	Fitted value	MAPE
BMI						
12	16.59	16.45	0.008	15.26	14.67	0.039
13	16.81	16.54	0.016	15.71	15.82	0.007
14	17.03	17.19	0.009	15.73	15.60	0.008
15	17.26	16.70	0.032	16.74	16.36	0.023
16	17.48	17.15	0.019	16.81	16.52	0.017
Average			0.017			0.019
Hand grip						
12	22.53	23.20	0.030	14.12	13.95	0.012
13	25.77	25.00	0.030	15.01	14.50	0.034
14	29.00	29.55	0.019	16.48	14.95	0.093
15	32.24	31.10	0.035	18.16	19.10	0.052
16	35.47	37.50	0.057	20.18	21.00	0.041
Average			0.034			0.046

MAPE, mean absolute percentage error.

The current study aimed to evaluate the health-related physical fitness of adolescents by establishing a normative standard using the LMS method and cross-validating the standard through a back substitution test to provide individual indicator indexes for our HRPF evaluation system.

### 3.1. Single index fitness evaluation criteria

#### 3.1.1. Method of establishing single indicator evaluation standard

In this study, the percentile method was adopted to formulate and constitute the rating standard for the evaluation system. This method was chosen due to the normal distribution of the samples for the two categories of indicators: anthropometric characteristics (BMI) and HRPF indicators. This approach allows for the appropriate interpretation and comparison of the results within the context of a normal distribution.

This study describes the index cut-off points for the five-level scoring system used in the current evaluation system. The evaluation of individual indicators utilizes a five-point scale, with scores ranging from 1 to 5. A score of 1 indicates poor performance, while a score of 5 indicates excellent performance. This five-grade classification method is utilized to determine an indicator’s relative position within the context of the entire age and gender group of the evaluated individuals. A higher score indicates better performance on a particular indicator. The scores for each indicator for both male and female ages specifically serve as the evaluation standard for that specific indicator.

After establishing LMS normative reference standard, the five-point index scoring standards are interpreted as follows: Index of anthropometric characteristics BMI categories “1 lean, obese: [≤P5, ≥ P95], 3 over-weight: [P85, P95), 5 normal weight: [P5, P85).” 1 represents a poor performance within P3–P10, 2 represents a below-average performance within P10–P35, 3 represents an average performance with in P35–P65, 4 represents a good performance within P65–P90, and 5 represents an excellent performance more than ≥P90. “1 Very Poor: [P3, P10], 2 Poor: [P10, P35), 3 Medium: [P35, P65), 4 Good: [P65, P90), 5 Excellent: ≥ P90.” This study has set a minimum limit line for individual score categories of HRPF indicators. If the test value is lower than the third percentile, no score will be given to encourage people to truly exercise their abilities in the test. No minimum line is set for the body shape test. Considering the characteristics of HRPF indicators in actual use.

#### 3.1.2. BMI index

The growth and development of adolescents are key indicators of their overall health status during this critical developmental period. One of the most visible manifestations of adolescent growth and development is the change in BMI concerning age. The results of the age and gender-specific BMI measurements are presented in Table 6. This information allows for the assessment of the adolescent’s growth and development status, as well as the identification of any potential health concerns.

**Table 6:** Index for BMI age and gender-specific according to the single indicator evaluation standard.

Indicators	Gender	Age	Lean	Normal Weight	Over Weight	Obese
BMI	Boys	12	13.08–14.32	14.33–19.14	19.15–20.81	≥20.82
		13	13.65–14.73	14.74–19.30	19.31–21.01	≥21.02
		14	14.09–15.07	15.08–19.52	19.53–21.34	≥21.35
		15	14.42–15.34	15.35–19.86	19.87–21.92	≥21.93
		16	14.69–15.58	15.59–20.26	20.27–22.67	≥22.68
	Girls	12	11.08–12.68	12.69–19.23	19.24–20.33	≥20.34
		13	11.58–12.82	12.83–18.27	18.28–20.40	≥20.41
		14	12.14–13.32	13.33–18.85	18.86–21.17	≥21.18
		15	12.99–14.25	14.26–18.97	18.98–21.58	≥21.59
		16	13.69–14.81	14.82–19.78	19.79–21.93	≥21.94

#### 3.1.3. Health-related physical fitness indicators single index

The current study has selected the hand grip strength and the modified pull-up test to evaluate adolescents’ muscular strength effectively. These primary indicators have been chosen for their relevance and accuracy in assessing muscular strength. The results of these evaluations are presented in Table 7, providing a comprehensive understanding of the age and gender-specific range Scores for hand grip and modified pull-up test for the adolescents of south Punjab region Pakistan.

**Table 7:** Indexes for HRPF indicators age and gender-specific according to the single indicator evaluation standard.

Indicators	Gender	Age	Very poor	Poor	Medium	Good	Excellent
Hand grip strength (kg)	Boys	12	8.14–11.56	11.57–18.63	18.64–27.03	27.04–40.21	≥40.22
		13	11.45–14.82	14.83–21.82	21.83–30.43	30.44–44.86	≥44.87
		14	14.55–17.94	17.95–24.98	24.99–33.82	33.83–49.31	≥49.32
		15	17.42–20.90	20.91–28.11	28.12–37.22	37.23–53.58	≥53.59
		16	19.96–23.63	23.64–31.17	31.18–40.66	40.67–57.81	≥57.82
	Girls	12	8.02–9.59	9.60–12.66	12.67–15.70	15.71–19.87	≥19.88
		13	7.51–9.69	9.70–13.24	13.25–16.90	16.91–21.81	≥21.82
		14	7.52–10.08	10.09–14.43	14.44–18.63	18.64–24.03	≥24.04
		15	8.81–11.63	11.64–16.14	16.15–20.23	20.24–25.23	≥25.24
		16	9.46–12.84	12.85–17.96	17.97–22.41	22.42–27.62	≥27.63
Modified pull-up (n)	Boys	12	1–2.58	2.59–6.98	6.99–12.51	12.52–21.29	≥21.3
		13	1–2.7	2.71–7.53	7.54–13.7	13.71–23.06	≥23.07
		14	1–2.83	2.84–8.24	8.25–14.64	14.65–23.56	≥23.57
		15	1–3.57	3.58–9.14	9.15–15.21	15.22–23.94	≥23.95
		16	3–4.78	4.79–9.77	9.78–16.37	16.38–27.52	≥27.53
	Girls	12	1–1.13	1.14–2.35	2.36–4.53	4.54–10.61	≥10.62
		13	1–1.16	1.17–2.39	2.40–4.63	4.64–11.87	≥11.88
		14	1–1.17	1.18–2.39	2.40–4.66	4.67–10.81	≥10.82
		15	1–1.17	1.18–2.41	2.42–4.72	4.73–10.68	≥10.69
		16	1–1.24	1.25–2.44	2.45–4.81	4.82–11.4	≥11.41
20 m shuttle run VO2_(MAX)_	Boys	12	33.64–35.84	35.85–44.21	44.22–47.53	47.54–50.18	≥50.19
		13	30.63–32.83	32.84–41.85	41.86–45.70	45.71–48.85	≥48.86
		14	29.11–31.31	31.32–39.59	39.60–43.86	43.87–47.58	≥47.59
		15	22.60–30.13	30.14–37.37	37.38–42.02	42.03–46.48	≥46.49
		16	24.64–29.06	29.07–35.23	35.24–40.20	40.21–45.71	≥45.72
	Girls	12	34.28–36.54	36.55–40.15	40.16–43.46	43.47–47.59	≥47.60
		13	33.07–34.83	34.84–37.96	37.97–41.32	41.33–46.40	≥46.41
		14	31.83–33.17	33.18–35.81	35.82–39.16	39.17–46.17	≥46.18
		15	30.18–31.24	31.25–33.56	33.57–37.28	37.29–38.40	≥38.41
		16	31.31–31.72	31.73–32.46	32.47–33.27	33.28–34.58	≥34.59
Plank exercise (s)	Boys	12	7.59–15.29	15.30–38.48	38.49–79.14	79.15–156.37	≥156.38
		13	8.27–15.40	15.41–38.86	38.87–77.91	77.92–161.88	≥161.89
		14	8.67–16.73	16.74–40.06	40.07–78.20	78.21–166.88	≥166.89
		15	12.88–20.45	20.46–41.72	41.73–78.57	78.58–168.61	≥168.62
		16	13.32–21.07	21.08–42.40	42.41–83.38	83.39–202.18	≥202.19
	Girls	12	8.50–14.20	14.21–33.15	33.16–54.38	54.39–83.63	≥83.64
		13	7.13–16.74	16.75–34.30	34.31–60.75	60.76–111.24	≥111.25
		14	10.93–17.71	17.72–36.82	36.83–69.66	69.67–148.01	≥148.02
		15	16.33–23.61	23.62–43.39	43.40–78.12	78.13–171.31	≥171.32
		16	17.43–25.86	25.87–49.07	49.08–90.00	90.01–197.90	≥197.91

The results of these evaluations, specifically those related to the 20 m shuttle run test, are presented in Table 7. This study provides extensive evidence to understand the adolescent’s cardiorespiratory fitness/aerobic capacity and allows for the identification of any potential areas of concern. The Plank exercise test was utilized to assess the level of muscular endurance in study participants. The findings of these assessments are outlined in Table 7, which presents a comprehensive analysis of the range of scores specific to age and gender, offering a deeper insight into the results of the Plank test. Table 7 describes indexes for HRPF indicators age and gender-specific according to single indicator evaluation standard range/scores for the 20 m shuttle run, hand grip, modified pull up, and plank for adolescents. As per the criteria, the single index system scoring classification method evaluation presents the hand grip, modified pull-up, plank exercise, and 20-m shuttle run test’s indexes (scores/cut-off) values for cardiovascular endurance/cardiorespiratory fitness, muscular strength, and muscular endurance.

### 3.2. Establishment of a comprehensive health-related physical fitness evaluation system

#### 3.2.1. Comprehensive evaluation index system

**Figure 3:**
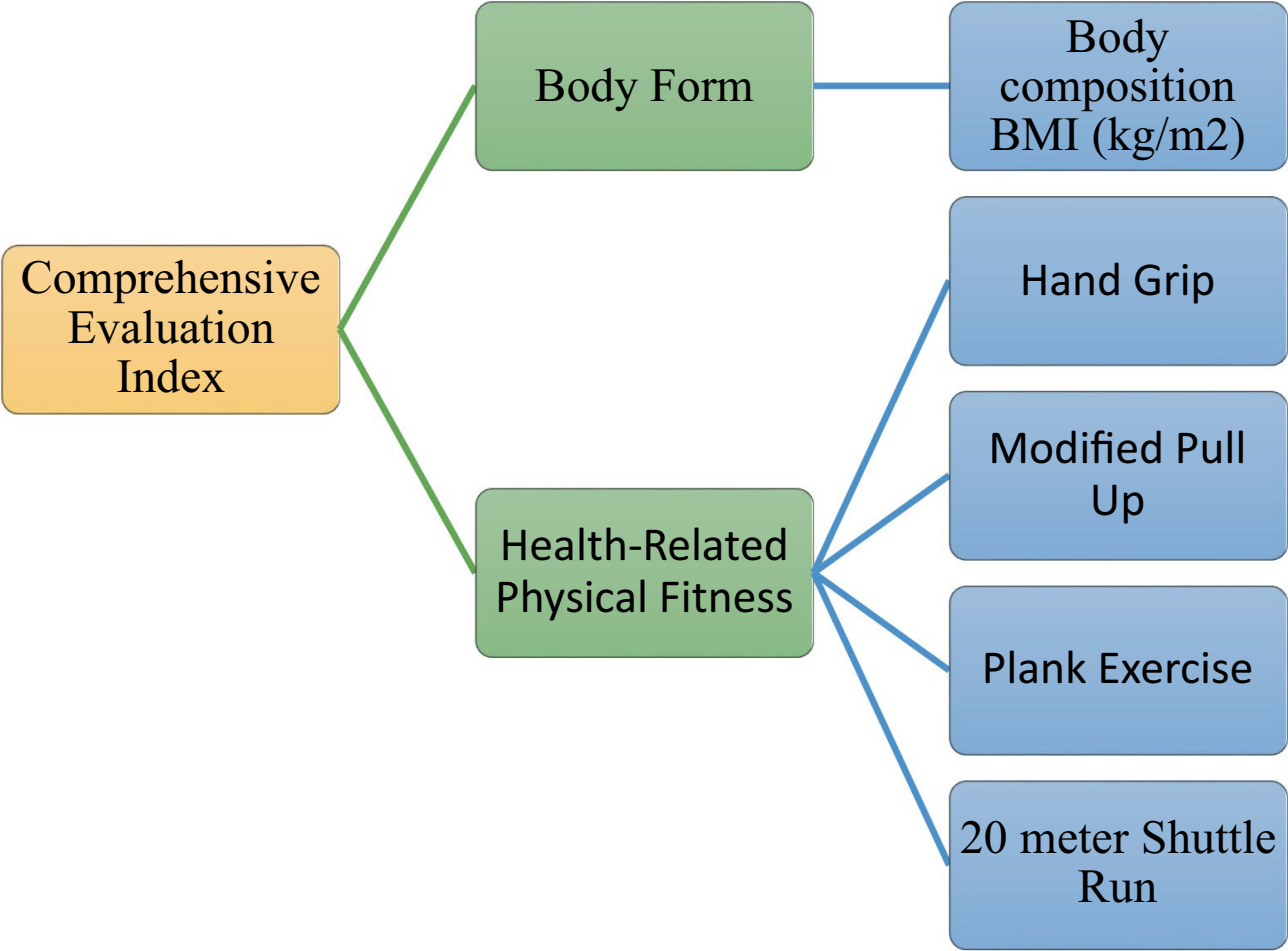
Comprehensive evaluation index system for school adolescents.

A comprehensive indicator system for assessing the growth and development of adolescents has been established (Figure 3) taking into account the nature of growth and development among the adolescent population of south Punjab’s region Pakistan, as well as the desirability, quantification, and representativeness of the indicators. Expert opinion was utilized to evaluate each indicator’s rationality and validity. The evaluation grade is divided into five categories: very reasonable, reasonable, basically reasonable, unreasonable, and very unreasonable, with scores of 1–5, respectively. Table 8 presents the statistical results of the expert evaluation. This approach ensures the selection of appropriate and relevant indicators for the assessment of adolescent growth and development. It allows for the identification of any potential areas of improvement in the indicator system.

**Table 8:** Inclusion statistics of rationality of each index in the HRPF evaluation system.

Number of questions	Frequency of each grade (score)					Less than 3 points Proportion
	1	2	3	4	5	
1	6	3	0	0	0	100%
2	8	0	1	0	0	100%
3	6	1	2	0	0	100%
4	5	2	2	0	0	100%
5	5	3	1	0	0	100%

In the process of further analysis method can be used to assess the relative importance of indicators by forming A judgment matrix (C_ij_) based on the five scales of relative importance. The matrix C_ij_ represents the relative importance of indicator C_i_ compared to indicator C_j_ for a specific level. By using a judgment matrix to measure the importance of indicators, it is possible to conduct a systematic and comprehensive analysis to determine the relative importance of each indicator for the specific level. This approach allows for the identification of key indicators that have the greatest impact on the level and guides decision-making on which indicators to prioritize in the evaluation process.


$$C_{ii}=C_{jj}$$

$$C_{ij}>0$$

$$C_{ji}=1/C_{ij}$$

The calculation process of indicator weight is as follows: build a judgment matrix C_ij_. Tables 9, 10 show from the evaluation results that the experts’ recognition of the health-related physical fitness indicator of adolescents reflected by their anthropometric characteristics (BMI) and HRPF has reached 100%, indicating that it is reasonable to choose BMI and HRPF indicators (HG, MPU, and PE and 20 m shuttle run test) as the first level indicators of the evaluation system of south Punjab region’s adolescents. Our results 100% of the experts believe it is reasonable to choose the body composition to reflect the body shape/anthropometric characteristics and HRPF indicators to reflect the overall health and fitness. The reasonableness of four secondary indicators of HRPF, including hand grip, modified pull-up, plank and 20 m shuttle run test, is relatively recognized, and the proportion of basically satisfied in each question (100%) is highly reasonable. At the same time, the experts have a good command of the hand grip, modified pull-up, plank, and 20 m shuttle run test.

**Table 9:** Comprehensive evaluation index judgment.

Indicators	Body composition/Shape	H Physical fitness
Body composition/Shape	1	3.44
H physical fitness	1/3.44	1

**Table 10:** Judgment matrix of health-related physical fitness indicators index.

Indicators	Hand grip	20 m SRT	Plank	Modified pull up
Hand grip	1	2.56	2.33	2.44
20 m SRT	1/2.56	1	2.56	3.89
Plank	1/2.33	1/2.56	1	3.56
Modified pull up	1/2.444	1/3.89	1/3.56	1

### 3.3. Weighting analysis and weight determination of all indicators for comprehensive evaluation according to expert opinion

According to the relative importance of each indicator, and the size of its impact on HRPF and role, determine their respective weight in the comprehensive evaluation system. Table 10 shows the Judgment Matrix of the HRPF index. The weight of each index is calculated according to the importance judgment matrix.

Table 11 shows the judgment matrix of comprehensive evaluation indicators and physical fitness indicators, respectively. According to the expert’s judgment Value of Index Importance statistic opinion analysis, all the indicators of health-related physical fitness and BMI were given weight in the evaluation system. Wherein, Equations (1) and (2) are weight calculation equations, and equations (3, 4) are weight logic error tests. The consistency test indexes are all less than 0.1, so it can be considered that the above matrices have good consistency.

**Table 11:** Weight of each evaluation index in the evaluation index system.

Major indicators	Weight coefficient	Index meaning	Secondary indicators	Weight
Body shape	28.07%	Body composition	BMI	28%
Health-related physical fitness	71.92%	Muscle strength	Hand grip	28.23%
		Cardio-respiratory fitness/Aerobic capacity	20-m shuttle run test	20.31%
		Core muscle endurance	Plank exercise	12.71%
		Upper muscular strength	Modified pull up	10.66%
Total	100%			100%

The formula is as follows:


(1) $$w_{i=W_{i}\sum_{i-1}^{m}w_{i}}$$

(2) $$W_{i}=\sqrt[{m}]{c_{i1}\times c_{i2}\times c_{i3}\times c_{im}}$$

(3) $$\lambda_{i=\sum_{i=1}^{m}C_{ij}W_{j}/W_{i}}$$

(4) $$C.I=\left(\lambda_{\text{max}}-m\right)\;\left(m-1\right)$$

The researcher made adjustments based on expert questionnaire suggestions, and the final weight of each indicator is shown in Table 11. According to the expert’s judgment Value of Index Importance statistic opinion analysis, all the indicators of health-related physical fitness and BMI were given weight by using above mention equation in the evaluation system as hand grip 28.23%, modified pull up 10.66%, plank 12.71%, 20 m shuttle run test 20.31%, and BMI 28.07% (Table 11). We compared the importance and unimportance of the indicators at the same level in the comprehensive evaluation system of adolescents according to their importance in the system and score.

### 3.4. Comprehensive evaluation criteria

The scoring standards for all indicators of adolescents in all age groups serve as the foundation for comprehensive evaluation. The final evaluation is determined by calculating the total score of all indicators based on the weights assigned to each indicator. This approach allows for a comprehensive assessment of the overall health status of adolescents by taking into account the various indicators that reflect different aspects of adolescent growth and development. The weighting system ensures that the most important indicators have the greatest impact on the final evaluation.

#### 3.4.1. Calculation formula of the total score


$$\mathrm{Total}\;\mathrm{score}=\left(\begin{array}{l} \mathrm{body}\;\mathrm{composition}\times 0.2807+20\;m\;\mathrm{shuttle}\;\mathrm{run}\;\\ Vo2_{\text{max}}\times 0.2031+\mathrm{grip}\;\mathrm{strength}\times 0.2823+\\ \mathrm{modified}\;\mathrm{pull}\;\mathrm{up}\times 0.1066+\mathrm{Plank}\;\mathrm{exercise}\times 0.1231 \end{array}\right)\text{.}$$

Example: The evaluation system was applied to a sample of 12-year-old male students, Wang. The results showed that the system effectively categorized their HRPF level based on their performance in the different physical fitness tests. Specifically, using the equation (1 × 0.2807 + 3 × 0.2031 + 2 × 0.2823 + 3 × 0.1066 + 4 × 0.1231), we calculated the total score for each participant, and the results were compared with Table 12 to determine their HRPF category. For instance, student Wang scored 2.247, indicating poor HRPF and placing him in the second category of the evaluation system. These findings demonstrate the potential of the proposed comprehensive evaluation system for monitoring and improving individual HRPF.

**Table 12:** Comprehensive rating criteria age and gender-specific score.

Categories	Score	Range
Very poor	1	<1.5
Poor	2	≥ 1.5 to < 2.5
Medium	3	≥ 2.5 to < 3.5
Good	4	≥ 3.5 to < 4.5
Excellent	5	≥ 4.5 to < 5

#### 3.4.2. Evaluation criteria

The evaluation process is divided into five grades: excellent grade, good grade, medium grade, poor grade, and very poor grade. The final evaluation grade is determined by calculating the total score of all indicators, considering the weight assigned to each indicator. Any indicator with no score will not be considered in the comprehensive evaluation. This approach ensures that only complete and accurate data is used in the evaluation process and eliminates any potential bias that may be introduced by missing data (Table 12). Comprehensive Rating Criteria score very poor, poor, medium, good, and excellent.

This study provides a health-related physical fitness evaluation system for adolescents in the South Punjab region of Pakistan. This evaluation system is categorized into five levels: very poor, pass, medium, good, and excellent. The HRPF evaluation system was used to assess the physical fitness level of the participants, where 17% of adolescents were categorized as poor, while 1.2% had a very poor HRPF level. About 50.7% of the adolescents had a medium HRPF level, and 24.9% were evaluated as good, whereas only 6.2% of the participants were classified as excellent.

The evaluation system generates age and gender-specific HRPF individual indexes, offering detailed research evidence to initiate effective strategies for improving the health of adolescents. These indexes provide an overview of adolescents’ HRPF levels, which can aid in developing appropriate intervention strategies for enhancing their physical fitness.

## 4. Discussion

It is the first time any information has been published on the evaluation system for adolescents’ health-related physical fitness in the south Punjab region of Pakistan. To the best of our knowledge, this is the first article to precisely examine HRPF indicator assessment with these indicators and tools in Pakistan. Although our study evaluated the physical fitness of adolescents in school settings, which may not be as accurate as laboratory tests with an epidemiological approach, assessments conducted in more experimental settings could provide a more realistic representation of real-world performance. Physical fitness, which is commonly understood as the basic ability of the human body to perform physical activity, is a reflection of the functions of various organ systems in relation to muscle work. Childhood and adolescence are crucial periods for the development of muscle strength, speed, and endurance. As such, the physical fitness evaluation during this time typically focuses on these key aspects to assess overall fitness and identify any areas of weakness or deficiency that may require further attention or intervention.

Muscular strength is a crucial component of HRPF that is often overlooked. Adolescents, in particular, must pay attention to their muscular strength levels to ensure healthy growth and development (20). Muscular strength plays a vital role in maintaining overall physical fitness and improving quality of life. Adolescents with adequate muscle strength are better able to perform daily activities and maintain good health. According to research, muscular strength positively correlates with physical performance and endurance (36). Strong muscles enable adolescents to engage in physical activities that require physical effort, such as playing sports, carrying heavy bags, and running long distances (37). In addition, adequate muscular strength helps to improve bone density and prevent the onset of chronic diseases such as osteoporosis (38). Studies show that physical activity, including resistance training, positively impacts bone mineral density (39). Adolescents who engage in regular physical activity that includes resistance training have an increased chance of maintaining strong bones throughout their lives.

Furthermore, muscular strength also plays a vital role in preventing obesity and reducing the risk of metabolic disorders such as diabetes (40). Adequate muscle mass and strength increase the body’s metabolic rate, which leads to the burning of more calories at rest. As a result, adolescents who have adequate muscle strength are less likely to become overweight or obese. The result of this study enables the identification of any potential areas of concern regarding their muscular strength, aiding in the development of targeted interventions to improve their overall health and well-being.

Assessing cardiorespiratory fitness/aerobic capacity is crucial to evaluating adolescents’ physical development and strength (41, 42). In selecting indicators for assessing cardiorespiratory fitness/aerobic capacity in adolescents, it is important to consider the overall development of the human body system and the ability to assess cardiorespiratory fitness/aerobic capacity during both rest and exercise. To this end, maximum oxygen uptake has been chosen as the primary indicator for effectively evaluating adolescents’ cardiorespiratory fitness/aerobic capacity. Cardiovascular fitness measures the body’s ability to use oxygen during physical activity. It is considered an important aspect of overall physical fitness, particularly among adolescents. Adolescence is a crucial development period during which physical, cognitive, and emotional changes occur. Adequate cardiorespiratory fitness is important for adolescents to be able to participate in physical activities, sports, and other forms of physical exertion without experiencing fatigue or shortness of breath. It also helps to improve their overall health and well-being. Adequate cardiovascular fitness is associated with a lower risk of cardiovascular diseases like hypertension, stroke, and heart disease (43, 44). Adolescents with ample cardiovascular fitness have a better physical function, including improved endurance and energy levels, allowing them to participate in physical activities and sports easily (45). Additionally, better cardiovascular fitness tends to have better mental health and academic performance, including a lower risk of depression and anxiety and improved concentration and cognitive function, respectively (46, 47).

Muscular endurance is one of the important components of health-related physical fitness, which refers to the ability of muscles to exert force repeatedly or sustain a contraction for an extended period (48). It plays a crucial role in maintaining good health and overall fitness, particularly among adolescents at a stage of growth and development. The plank test is one of the commonly used measures of muscular endurance that can be used to assess and monitor the fitness level of adolescents (33, 49, 50). The plank test is a simple and effective method of assessing the muscular endurance of the core muscles, including the rectus abdominis, transverse abdominis, and erector spinae muscles (51, 52). The plank test has several benefits in assessing muscular endurance among adolescents. It is a quick and easy test that does not require special equipment or expensive technology, making it accessible to individuals of all fitness levels (51, 52). It is a safe and non-invasive method of assessing fitness, as it does not involve strenuous or potentially harmful exercises. The plank exercise test is an objective and reliable measure of muscular endurance that quantifies how much time an individual can hold the plank position. It can help identify individuals at risk of developing musculoskeletal problems due to poor core stability, such as lower back pain or postural abnormalities (53, 54). It can motivate adolescents to improve their fitness level and overall health by setting achievable goals for increasing their plank time (27, 55). Lastly, the plank test can help to promote good posture and balance, which are important for preventing falls and maintaining proper body alignment (56). The period of adolescence presents an opportune time for the development of muscular endurance due to the heightened excitability of the cerebral cortex and the adaptability of neural processes. Table 8 shows the health-related physical fitness evaluation system individual indexes age-specific and gender-specific for adolescents in the South Punjab region of Pakistan. This evaluation system is categorized into five levels: very poor, pass, medium, good, and excellent. The results of the HRPF evaluation system was used to assess the physical fitness level of the participants, where 17% of adolescents were categorized as poor, while 1.2% had a very poor HRPF level. About 50.7% of the adolescents had a medium HRPF level, and 24.9% were evaluated as good, whereas only 6.2% of the participants were classified as excellent. This data provide an overview of the HRPF level of adolescents in the region and can be useful for developing appropriate intervention strategies to improve the physical fitness of this population.

Additionally, adolescents in this age range may lack knowledge and coordination when participating in HRPF assessments. It is also worth noting that the flexibility component of HRPF was not included in this study, following the United States committee on fitness measurement and health outcomes in the adolescent recommendation, which concluded that the relationship between flexibility and health was unclear due to the lack of scientific literature (57). Therefore, flexibility was not considered in the current study.

Although our study evaluated the physical fitness of adolescents in school settings, which may not be as accurate as laboratory tests with an epidemiological approach, assessments conducted in more experimental settings could provide a more realistic representation of real-world performance.

## 5. Conclusion

This study successfully developed a foremost systematic health-related physical fitness evaluation system to assess the health of adolescents in the South Punjab region of Pakistan. The study filled a research gap by providing an evaluation system for HRPF in Pakistan. This study also established the index of two primary indicators, BMI and four HRPF indicators. The weight coefficients were determined by the opinion of the experts for both the primary and secondary level indicators, resulting in a comprehensive evaluation standard. This system provides precise age and gender-specific HRPF assessments, identifying potential areas of concern. With the application of this evidence, effective interventions can be designed to address these health issues. Therefore, this study contributes significantly to the academic literature and is a valuable tool for public health professionals in promoting the health and well-being of adolescents in Pakistan.

## Data availability statement

The raw data supporting the conclusions of this article will be made available by the authors, without undue reservation.

## Ethics statement

The studies involving humans were approved by The Ethics Advisory Committee of the School of Exercise and Health at the Shanghai University of Sport. The studies were conducted in accordance with the local legislation and institutional requirements. Written informed consent for participation in this study was provided by the participants’ legal guardians/next of kin.

## Author contributions

SMZHH and JZ contributed to conception and design of the study. SMZHH, SGH, SDHH, SD, and SF organized the database. SMZHH and HK performed the statistical analysis. SMZHH wrote the first draft of the manuscript. All authors contributed to the article and approved the submitted version.
